# Motor Unit Firing Behaviour of Soleus Muscle in Isometric and Dynamic Contractions

**DOI:** 10.1371/journal.pone.0053425

**Published:** 2013-02-06

**Authors:** Jouni Kallio, Karen Søgaard, Janne Avela, Paavo V. Komi, Harri Selänne, Vesa Linnamo

**Affiliations:** 1 Neuromuscular Research Center, Department of Biology of Physical Activity, University of Jyväskylä, Jyväskylä, Finland; 2 Institute of Sports Science and Clinical Biomechanics, University of Southern Denmark, Odense, Denmark; 3 Neuromuscular Research Center, Department of Biology of Physical Activity, University of Jyväskylä, Jyväskylä, Finland; 4 Neuromuscular Research Center, Department of Biology of Physical Activity, University of Jyväskylä, Jyväskylä, Finland; 5 LIKES Foundation for Sport and Health Sciences, Jyväskylä, Finland; Universidad Europea de Madrid, Spain

## Abstract

**Introduction:**

Understanding the detailed control of human locomotion and balance can be improved, when individual motor units can be isolated and their firing rates followed in natural movement of large, fuctionally important muscles. For this reason the present study investigated the motor unit discharge rate (MUDR) in isometric and dynamic contractions of the soleus muscle. Methods: Eleven males performed isometric (10–100% MVC) and dynamic (10–40% MVC) plantar flexions. Intramuscular EMG was measured from Soleus with bipolar wire-electrodes and decomposed with custom built “Daisy” software.

**Results:**

The Soleus MUDR was significantly higher in concentric compared to isometric or eccentric contractions at all submaximal force levels (P<0.05). In isometric contractions MUDR increased up to 100% MVC. Conclusion: Motor unit discharge properties of a large plantarflexor can be measured in dynamic and maximal contractions. For a given torque output, MUDR is dependent upon contraction type, as set by the major mechanical differences between concentric and eccentric actions.

## Introduction

It is well known that to produce a certain force requires less motor unit activation in eccentric as compared to concentric action [1]. This has been clearly demonstrated for both upper arm and lower leg muscles (e.g. [2]–[11]. Compared to isometric conditions, eccentric contractions are associated with lower reflex excitability [12], [13]. Contraction type has also been shown to relate to different motor unit activation patterns, like synchronization [14], while changes in motor unit recruitment order seem to be small or non-existent (for review see: Chalmers 2008 [15]).

Due to the challenges of recording during dynamic contractions, most studies investigating single motor unit activation have concentrated on isometric contractions. The few earlier studies comparing muscle contraction types in dynamic movements have mostly been performed in upper extremity muscles and have shown the largest motor unit discharge rates (MUDR) in concentric compared to eccentric and isometric contractions. These studies examined the elbow flexors [7], [9], [10], [16] and extensors [17], wrist flexors [18], the first dorsal interosseus [8], [19], and these results were confirmed in the studies on the knee extensors [20] and Tibialis Anterior [6].

In order to better understand the mechanisms of human movement it is important to investigate motor unit activation also in the large plantar flexors. The soleus muscle plays an important role in locomotion and balance control [21], [22] and seems to be modulated bilaterally, to some degree, by common drive in quiet standing [23]. As it has been shown that for example age-related differences in balance are more pronounced in dynamic conditions than in static stance [24], it is of importance to extend the research on the soleus muscle motor unit control to dynamic tasks. As soleus MUDR has only been studied in isometric contractions (e.g. [25]–[29], the main purpose of the present study was to identify possible differences in Soleus motor unit discharge rates between isometric, concentric and eccentric contractions. This is possible by utilizing highly selective wire-electrodes that enable the recording of single motor units from a large muscle with a high number of motor units and a large range of movement in dynamic contractions. In addition, the aim was to investigate the possibility of recording MUDR's up to maximal contractions with wire electrodes that enable performance of dynamic contractions.

## Materials and Methods

### Subjects

11 physically active males (26.1±2.2 yr. 1.81±0.05 m 81.7±9.4 kg) volunteered as subjects for the study. Subjects' physical activity and health were assessed with a questionnaire. Only subjects without any history of neuromuscular or vascular disease were approved for the study.

Full advice about possible risks and discomfort was given to the subjects and they all gave their written informed consent to the procedures prior to the commencement of the study. The investigation was conducted according to the declaration of Helsinki and approved by the ethics committee of the University of Jyväskylä.

### Protocol

The subjects performed isometric and dynamic plantar flexions while seated in a custom made ankle dynamometer (University of Jyväskylä, Finland). Prior to data collection all subjects performed a warm-up consisting of 10 minutes light cycling on a bicycle ergometer, 5–10 submaximal and for practice 5 maximal isometric plantar flexions. All measurements were conducted in relation to the individual range of motion of the subject. In order to determine the individual zero angle (IZA), the voluntary range of motion was determined according to the protocol of [30]. Subjects were asked to perform slow maximal dorsi and plantar flexions in the ankle dynamometer without external/additional load. The range of motion was calculated as the difference between the two maximum angles. IZA was determined by subtracting 15 degrees from the maximal voluntary dorsiflexion angle. On average the IZA was 87.6±1.8 degrees. All isometric contractions were performed at each subject's IZA and with a fixed straight knee (180 deg).

**Figure 1:**
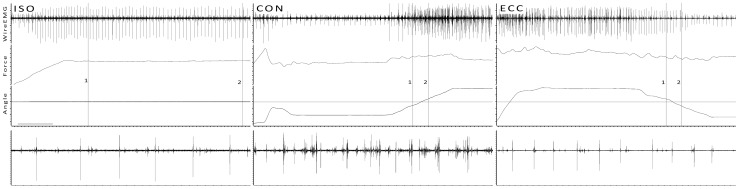
Examples of ISO, CON and ECC trials from one subject at 10% MVC. TOP: Cursors 1 and 2 mark the beginning and end of analysis, respectively. In dynamic contractions Cursor 1 is 200 ms prior and Cursor 2 200 ms after the angle crosses the angle of the ISO trial, as indicated by the horizontal cursor. BOTTOM: 400 ms close-ups of the wire EMG signal of each trial.

The test battery was as follows:

1. Maximal voluntary contractions (MVC)The subject was instructed to perform a maximal isometric plantar flexion for approximately 5 s. A minimum of two trials were performed and further trials were performed if the maximal force value between trials differed by more than 10%. The MVC measurement was repeated at the end of the protocol to examine the extent of possible fatigue caused by the session. A three minute rest was given between successive MVC trials.
2. Submaximal trials
  - Isometric contractions (ISO): The force levels used in the contractions were 10, 20, 40, 60, 80 and 100% of the isometric MVC. The target force level was displayed on the monitor for the subjects to match. The subjects were instructed to increase the force to this level and hold it as steady as possible for several seconds, ranging from 10 s at 10 & 20% MVC to a couple of seconds at 100% MVC. Analysis was done from a time-window when the force was stationary and on target. Each subject practiced the task several times before the insertion of wires.
  - Dynamic contractions: Dynamic contraction measurement tasks can be performed in different setups either by controlling the force variation as the limb is moved with a constant velocity by an isokinetic machine or controlling the movement velocity against a constant inertial load. The present study used the latter setup, as the subjects lifted (concentric: CON) or lowered (eccentric: ECC) a weight stack that was attached to the foot pedal via a cable pulley system at a voluntarily controlled velocity of 10 deg/s. The weight was adjusted so that the ankle torque during movement was identical to the corresponding ISO condition. The submaximal force levels in dynamic measurements corresponded to 10, 20 and 40% of the isometric MVC. In some subjects also higher dynamic contractions were measured, but due to the low number of successful trials with identifiable units in the highest levels, only the lowest three levels are reported here. The subjects controlled the movement velocity by guidance from visual feedback of the ankle angle on a monitor that had cursors set for both the correct start and end times and the start and end angles of the movement. The range of motion was set to enable a measurement window clearly separated from the acceleration or deceleration phase of the movement. Analysis was done from a 400 ms window around the ISO angle (Figure 1). Both angular velocity and torque were analyzed online and a trial was only included in the study if both of the values were within 2.5 percentage points of the target.

### Force and surface EMG

Trials were performed while subjects sat with the foot attached to an ankle dynamometer pedal mounted with torque (HPM, Germany) and angle (Vishay 357-0-0-103, USA) sensors. To maximize stability during testing, subjects were strapped to the chair, the chest with a 4-point seatbelt and the thigh and ankle with straps. The foot pedal was attached to a weight stack via a pulley. The measured torque signal was sent to a computer via an A–D board CED 1401+ (Cambridge Electronic Design) and sampled at a frequency of 50 kHz. Signals were collected and analyzed using Signal software (Cambridge Electronic Design, UK). Before analysis, force and angle signals were downsampled to 2 kHz, and then filtered with a low-pass filter (cutoff frequencies 75 and 150 Hz, respectively).

Global EMG activity of the soleus (SOL) and gastrocnemius medialis (GM) muscles was recorded using bipolar silver chloride miniature surface electrodes (Beckman 650437, USA) with 20 mm inter-electrode distance. Electrodes were prepared and placed in accordance with the recommendations by SENIAM [31]. The surface EMG (sEMG) signals were amplified and sampled at a frequency of 50 kHz. The signals were subsequently downsampled to 2 kHz, band-pass filtered at 20 to 1 kHz and analyzed using Signal software (Cambridge Electronic Design, UK). Surface EMG activities of SOL and GM were calculated as root mean square (RMS) of the signal. For the maximal voluntary contractions (MVC), both sEMG and torque data were analyzed ±100 ms around the peak torque.

### Intramuscular EMG

For the intramuscular EMG recordings, four separate bipolar fine-wire electrodes were inserted into the soleus muscle. This provided four potential signals that could be used for the decomposition. Electrodes were inserted to soleus from the lateral side of the sEMG electrode at different depths (approximately 10-25 mm), and a ground electrode was placed on the medial malleolus. The wire-electrodes consisted of two 50 μm polyurethane insulated wires that were glued together. Insulation was mechanically removed from the outer sides of the wires over 0.5 mm distance from the tip, resulting in an inter-electrode distance of approximately 100 μm. A hook was formed at the end of the wires to ensure fixation during the measurements. Electrodes were autoclaved before the experiment in order to minimize the risk of infection. The wire electrode pairs were inserted into the muscle with a 22 gauge needle that was subsequently withdrawn. Wire-electrodes were inserted after the MVC trials to avoid possible electrode migration and/or loss of signal due to MVC force levels. Further, all measurements were started with the lowest force-levels to secure data from submaximal levels before risking the possible breaking at the largest force levels. In some cases a wire stopped recording during the measurements for an unknown reason. To optimize signal quality, the signal was pre-amplified with a non-commercial amplifier (amp 200, bandwidth 8–4500 Hz) attached to the calf of the subject. The amplified signal was sent to a CED 1902 programmable signal conditioner (Cambridge Electronics Design Ltd, Cambridge, UK) and digitized with CED 1401+ (Cambridge Electronic Design) with a sampling frequency of 20 kHz. Since the decomposition software allows visualization of three channels, the wire channel was duplicated twice and the two new channels were filtered with different fourth order butterworth band-pass filters (300–8000 Hz and 400–10000 Hz). Observing three differently filtered channels gave the analyzer more information for decomposition.

Signal decomposition, motor unit identification and data analysis were performed by utilizing the three channel decomposition technique computer algorithm, “Daisy”, described in more detail in [9], [32], [33]. Motor unit action potential (MUAP) recognition is based on template matching of the waveform of the three channels (unfiltered and two band-pass filtered), created from the same intramuscular EMG recording. The MU classification was performed semi-automatically with a high degree of operator interaction. The identification is based on the definition of templates from the three channels. The templates were updated as a running average of the last 10 detected MUAPs, allowing for the unavoidable gradual amplitude and shape changes of the MUAPs due to slight changes in the position of the wire relative to the detected unit. All firings were checked or sorted manually via visual inspection, and only the units with either a stable or a gradual and systematical change in shape and amplitude were included for further analysis. For each train of MU discharges the inter-spike intervals were registered and instantaneous motor unit discharge rate (MUDR) for each MU was calculated as the mean of the inverse of each inter-spike interval. The decomposed units from all subjects were pooled for calculating the differences between contraction types and levels. In addition, units that could clearly be recognized in at least two of the three contraction types were analyzed in order to investigate the firing rate behavior in more detail. Analyses were checked by comparing MUDRs to eliminate the inclusion of the same motor unit by more than one bipolar wire set.

### Statistical analysis

The normality of distribution was tested with the Shapiro-Wilk test. In the pooled units an analysis of variance (ANOVA) was used to compare the effects of contraction types and intensities, while repeated measures ANOVA was used for the units recognized in more than one condition. When a significant difference was detected, Bonferroni's method was used to locate the difference. The critical level of significance was P<0.05. Descriptive statistics include mean and standard deviation.

## Results

### Submaximal force levels

Submaximal force levels were compared between contraction types and no consistent or statistically significant differences were found at any of the force levels (Table 1). Furthermore, MVC before and after was not significantly different indicating that fatigue did not develop during the experiment.

**Table 1:** Number of subjects and motor units (MU), motor unit discharge rate (MUDR) and relative force and sEMG levels of soleus (SOL) and gastrocnemius medialis (GM) in different conditions.

Type	Level	N(subj)	N(MU)	MUDR (1/s)			Force%			SOL%			GM%		
**ISO**	**10**	10	43	7.3	±	2.2^†^	8.8	±	0.9	10.5	±	3.9^†‡^	6.2	±	4.2^†‡^
	**20**	10	44	8.3	±	2.2*^†^	17.7	±	0.7	16.5	±	4.8*^†‡^	7.4	±	2.6^†‡^
	**40**	8	45	9.1	±	2.2^†^	34.9	±	1.5	31.3	±	10.3*	17.3	±	7.6*^†‡^
	**60**	8	30	10.4	±	2.2*	55.7	±	6.5	46.3	±	14.0*	27.3	±	13.2*
	**80**	5	12	13.5	±	2.6*	74.7	±	7.0	63.7	±	17.4*	60.0	±	33.4*
	**100**	5	8	19.9	±	7.3*	99.4	±	6.1	91.6	±	20.2*	102.4	±	32.2*
**CON**	**10**	9	38	10.1	±	3.0	9.4	±	1.3	22.2	±	8.0	9.6	±	3.9
	**20**	8	16	11.6	±	2.3	19.2	±	0.7	34.0	±	7.6*	14.4	±	4.6*
	**40**	3	5	12.2	±	2.2	37.7	±	0.5	51.4	±	2.9*	41.6	±	15.4*
**ECC**	**10**	7	18	6.9	±	2.4^†^	9.6	±	1.3	13.9	±	3.9^†^	11.7	±	3.8
	**20**	9	20	8.2	±	2.3^†^	20.8	±	1.9	21.7	±	7.8*^†^	22.8	±	10.2*
	**40**	4	6	7.6	±	0.7^†^	38.5	±	1.8	32.7	±	10.0*^†^	37.1	±	13.8

Force and sEMG levels are calculated relative to isometric MVC. *: significantly larger than in lower force level. ^†^ : significantly smaller than in CON. ^‡^ : significantly smaller than in ECC (P<0.05).

### Motor unit discharge rate

The analysis was based on 5773 motor unit firings from 187 different motor units. The number of subjects with decomposable units in each condition ranged between 3 and 10, and the number of units between 5 and 45 (Table 1).

**Figure 2:**
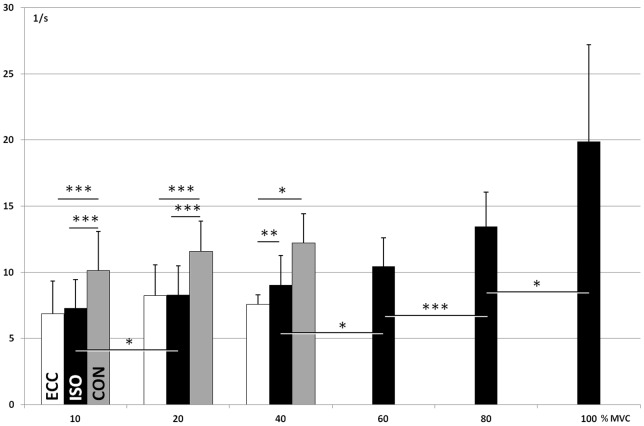
Motor unit discharge rate (1/s; X±SD) at different relative force levels (%MVC) in eccentric (ECC), isometric (ISO) and concentric (CON) contractions. Significant differences between conditions and force-levels: *  = p<.05, **  = p<.01, ***  = p<.001.

In ISO contractions, where force levels were explored up to 100% MVC, the MUDR increased significantly with force. The increase followed the relative force levels (Figure 2, Table 1). The MUDR increased significantly from 10 to 20, 40 to 60, 60 to 80 and 80 to 100% MVC. The increase followed a second order polynomial equation y = 0.64x^2^ –2.23x+9.43 (R^2^ = 0.9766).

With increasing force new units were recruited, with generally lower initial firing rates compared to units that were already active at lower force levels. Therefore the average difference in MUDR between force-levels was always larger within MU pairs when compared to the average of all analyzed units (table 2).

**Table 2:** The difference in motor unit discharge rate (MUDR) between force levels in motor unit pairs (top) and all pooled units (bottom) in isometric contractions.

Level1	Level2	N	MUDR 1			MUDR 2			MUDR diff%
		Pairs							
10	20	21	7.6	±	2.1	9.3	±	1.9	23.2*
20	40	15	7.9	±	1.9	10.0	±	2.4	25.5*
40	60	9	8.9	±	2.1	11.4	±	1.9	28.5*
60	80	5	8.7	±	0.5	11.8	±	0.7	34.3*
		Pooled		±			±		
10	20	43/44	7.3	±	2.2	8.3	±	2.2	13.7*
20	40	44/45	8.3	±	2.2	9.1	±	2.2	9.6*
40	60	45/30	9.1	±	2.2	10.4	±	2.2	14.3*
60	80	30/12	10.4	±	2.2	13.5	±	2.6	29.8*

* : significant difference (P<0.05).

**Figure 3:**
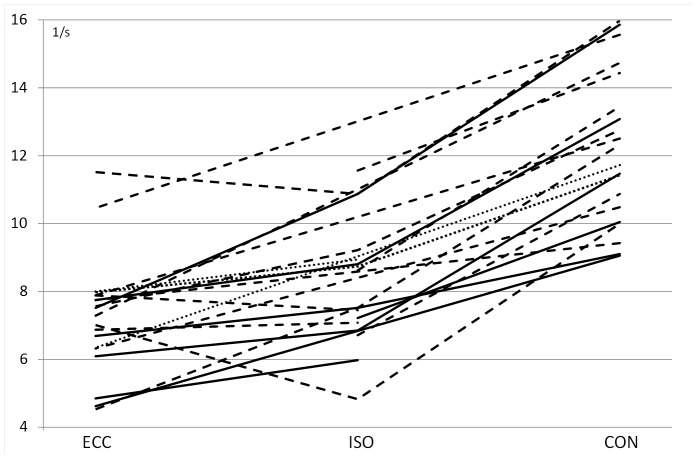
Discharge rates of motor units that could clearly be recognized in at least two of the three contraction types at 10 (solid line), 20 (dashed line) and 40% of MVC (dotted line).

At the three lowest force levels all three contraction types were measured and showed that the MUDR was significantly effected by contraction type. MUDR was significantly higher (p<.05) in CON compared to ISO or ECC, regardless of whether the comparison was based on all analyzed units (Figure 2, Table 1) or only on units that could clearly be recognized in at least two of the three contraction types (Figure 3). No significant differences in MUDR were found between ISO and ECC in either of the comparison methods. Of the 14 MUs recognized in both ISO and ECC, 9 fired with a higher discharge rate in ISO and 5 in ECC.

**Figure 4:**
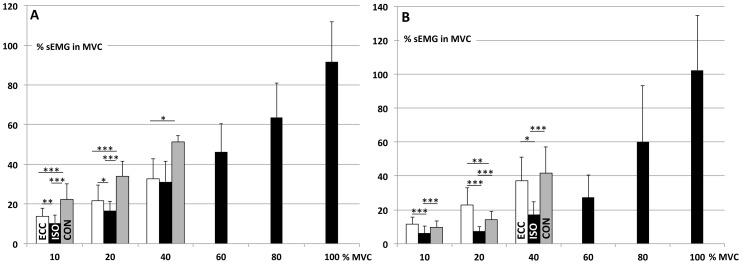
Relative muscle activity in different trials. sEMG levels of soleus (A) and gastrocnemius medialis (B) at different force levels in eccentric (ECC), isometric (ISO) and concentric (CON) contractions. sEMG is expressed relative to maximal isometric contraction (MVC). Significant differences between conditions and force-levels: *  = p<.05, **  = p<.01, ***  = p<.001.

The global sEMG activity of Soleus, as measured by the surface electrode, increased with increasing relative force in all contraction types (Figure 4A). In general, concentric contractions required more activation than ISO or ECC and at the two lowest force levels sEMG was significantly higher in ECC than in ISO. In gastrocnemius the relative sEMG also increased with increasing force (significant in all conditions except ISO 10–20% and ECC 20–40%). However, in this muscle the smallest sEMG was measured in ISO (Figure 4B).

## Discussion

The main result from the present study is that the soleus motor unit discharge rate was found to 1) increase with activation level up to 100% MVC and 2) be highest in concentric contractions in all measured submaximal trials when absolute torque levels were matched between contraction types.

### Isometric contractions

We found the average soleus MUDR to increase with increasing torque level from 7.3±2.2/s at 10% MVC to 19.9±7.3/s at MVC. The average maximal MUDRs were slightly higher than those reported in soleus by Dalton et al. 2010[25] (16.4±5.3/s) and almost twice the values of Bellemare et al. 1983 [27] (10.7±2.9/s). The differences between our results and those of Bellemare's study could be partly due to a higher average subject age (30–55 vs. 26±2 yr), as MUDR decreases with aging in Soleus [34] and this age-related difference is largest for maximal contractions (for review see Roos et al. 1997 [35]). The current data adds support to the suggestion that soleus force control works with a much more limited range of motor unit discharge rates compared to most other studied muscles, like adductor pollicis (30/s), biceps brachii (31/s), tibialis anterior (33/s) and abductor digiti minimi (52/s) [27], [36], [37].

The torque-related increase in MUDR followed a second order polynomial equation with a steeper MUDR increase at higher torque levels. In our earlier intramuscular EMG study [4] the increase in MU activity at moderate activation levels from 20 to 40% EMG at MVC was more pronounced in intramuscular mean spike amplitude than in mean spike discharge frequency, indicating a stronger contribution from motor unit recruitment compared to rate coding. However, at high force levels it is likely that increases in Soleus force rely more on an increase in MUDR than MU recruitment, even though some units have been observed to have thresholds above 90% MVC [26].

### Dynamic contractions

On average motor units were recorded at the same ankle angle in all conditions, although the start and end angles in dynamic conditions differed from ISO. Thus, in ECC for example, the possible effect of shorter fascicle length in MUDR at the start of recording would be cancelled by the adversary effect of a longer fascicle in the end position. Several studies have shown fascicle length to be quite independent of total muscle-tendon length in dynamic movements, like jumping [38], walking, running [39] and isokinetic maximal contractions [40]. We did not measure fascicle length directly, but as average joint angle and force were equal in ISO, CON and ECC, average fascicle length can also be assumed to be the same. This suggestion is supported by the study by Pasquet and colleagues [6], showing that the fascicle length change in tibialis anterior varied linearly with ankle joint angle.

A quite modest joint rotation velocity (10 deg/s) was chosen in order to have a sufficiently wide time window for the analysis. Even at this slow pace, the analysis was on average based on only 6.6 firings per trial. However, in order to reduce the error, the MUDR calculation included only the inter-firing-intervals of the firings that were detected within the measurement window (not the time before the first and after the last firing). The rotation velocity was quite slow compared to most natural movements. For example, in walking, the angular velocity in dorsiflexion is ∼30 deg/s and in plantarflexion ∼220 deg/s (estimated from Lichtwark 2007 [41]). Therefore this study is a step in the direction of investigating how the motor unit activation functions in normal locomotion.

The present study showed a larger MUDR in CON compared to ISO or ECC at low submaximal force levels. Our previous experiments [4] also showed the intramuscular mean spike frequency (an indirect measure of MUDR) to be slightly higher (n.s.) in CON than in ISO or ECC when different contraction modes were matched by surface EMG. In the current measurements the difference in the required neural input to produce equal torque may have amplified the difference in MUDR.

Two possible reasons for the lower MUDR in ECC have been suggested previously [42]. Firstly, mechanically, less torque is required to lower (ECC) the same absolute load compared to lifting it (CON). However, in the present study we controlled the weights so that the weight in CON was lower than in ECC, making the absolute plantarflexion torque in all contraction types the same. Secondly, the relative load in ECC contraction may be lower due to the higher maximal torque capacity in ECC. It is likely that the cross-bridges generate more torque when resisting pull (ECC) than they do when producing movement (CON) [43]. We measured MVC only in ISO, so the dynamic torques relative to their corresponding maxima are unknown. In an earlier study on plantarflexors the difference between MVCs in CON and ECC was 17% [44]. In the present study the difference in MUDR between CON and ECC at the three force levels was between 29–38%. It thus seems that the difference between contraction types is larger in MUDR than absolute torque production. This is in accordance with the study of [20] which showed a higher MUDR in CON even when force-velocity-related differences in force capacity were taken into account.

Surface muscle activity of soleus (sEMG_SOL_) behaved similarly to MUDR in different measurement trials. Both MUDR and sEMG_SOL_ were largest in CON, however only sEMG_SOL_ increased with torque levels in dynamic contractions. The torque related increase in neural drive to the muscle is probably more visible in sEMG_SOL_ due to motor unit recruitment. At the lowest activation levels, new, slowly firing units were recruited as force level was increased. This would have a diminishing effect on the average MUDR, while the recruited units would still increase the amplitude in sEMG_SOL_ [45].

The behaviour of gastrocnemius activity differed from soleus in the dynamic contractions, as the gastrocnemius activity in ECC was as at the same level as in CON. This phenomenon has previously been reported by Nardone and colleagues [46] and could reflect the more dominant role of gastrocnemius in normal dynamic contractions. In two classic papers, Nardone et al. reported the shift to a faster muscle (gastrocnemius) and the EMG findings as evidence for reversal of the recruitment order of motor units [46], [47]. With equal torques, an increase in gastrocnemius activity could remove some of the load from soleus. In the present study 9 of the 14 units that could be followed in both ECC and ISO showed a lower DR in ECC while in 5 units the DR was unaltered or higher in ECC. It therefore seems that the motor unit rate coding in ECC varies more than in CON, where the MUDR was always higher than in the other recognised conditions. It is possible that the increased activation of gastrocnemius affected the soleus activation more in recruitment than in rate coding. Recruitment threshold has also been observed to be lower in nonisometric contractions [7], [48]. In the present study the focus was on the firing rate of a random sample of motor units and thus we did not analyze recruitment order systematically. However, in ECC trials we observed several clear cases of derecruitment of units that had been active in ISO and/or CON, but none vice versa. This is in line with a number of other studies on dynamic contractions (for review see Chalmers [15]).

### Conclusion

Soleus motor unit discharge rate was found to be highest in concentric contractions in all measured submaximal trials when absolute torque levels were matched between contraction types. Being able to measure functionally important extensor muscles at maximal levels and in dynamic contractions offers possibilities for future research in various contexts, such as natural locomotion, dynamic balance control and ageing.
